# The Effect of Position on Radiographic Angle Measurements of the Lower Extremities

**DOI:** 10.1155/2022/1057227

**Published:** 2022-03-07

**Authors:** Jeehyeok Chung, Joonhee Lee, Hyuk-Soo Han, Myung Chul Lee, Du Hyun Ro

**Affiliations:** ^1^Department of Orthopedic Surgery, Seoul National University Hospital, Seoul, Republic of Korea; ^2^Seoul National University College of Medicine, Seoul, Republic of Korea; ^3^CONNECTEVE Co., Ltd, Republic of Korea

## Abstract

**Purpose:**

Accurately measuring an angle on a lower extremity X-ray is essential for the diagnosis and treatment of knee osteoarthritis (KOA). However, the angle is often affected by position, especially with flexion contracture and rotation. To date, there have been no quantitative analyses examining the relationship between lower extremity angle and patient position and no studies targeting patients with deformities. The aim of this study is to quantify the effect of position on angle measurements in lower extremity X-rays and to compare the effect in patients with different deformities.

**Methods:**

Computed tomography (CT) data of 131 patients with knee pain were retrospectively analyzed. The subjects were categorized into the following groups: neutral (hip-knee-ankle angle (HKAA) between 175 and 185°), varus (HKAA less than 175°), valgus (HKAA more than 185°), and flexion (flexion contracture more than 10°). CT images were digitally reconstructed to anterior-posterior X-ray images using an average intensity projection algorithm. The process was then repeated while rotating the reconstruction plane from internal 9° to external 9°. In this manner, X-ray images were reconstructed in different rotational states. The following angles were measured from reconstructed X-ray images: HKAA, lateral distal femoral angle (LDFA), medial proximal tibial angle (MPTA), and femoral valgus angle (FVA). The measurements were then compared according to the degree of rotation.

**Results:**

FVA significantly differed according to rotation in all groups (*P* < 0.001), with a difference of 1.3° (±0.4°). HKAA significantly changed only in the flexion contracture group (*P* < 0.001), which showed a difference of 1.0° (±0.7°). However, HKAA in the other groups, LDFA, and MPTA did not significantly differ depending on rotation.

**Conclusions:**

Radiographic measurement of FVA is subject to change according to rotation. HKAA significantly changed only in the flexion contracture group, so more care should be taken while obtaining X-rays of patients with flexion contracture.

## 1. Introduction

Measuring lower limb alignment angles using X-rays plays an important role in the diagnosis and treatment of knee osteoarthritis (KOA) [1–3]. Varus alignment with decreased medial proximal tibial angle (MPTA) is indicated for high tibial osteotomy if the patient complains of knee pain. Valgus alignment with decreased lateral distal femoral angle (LDFA) may be indicated for distal femur osteotomy. For knee surgeons, measuring these angles is part of the daily routine in the clinic.

However, it is often the case that these angles change for no apparent reason. These angular changes are explained away as the result of technical errors arising from the distance from the cassette or X-ray beams, the parallax effect of the X-ray beams, and the position of the lower extremity [4–13]. Positioning the patient such that the patella faces forward, which is common while taking radiographs, may put the lower extremity in different rotational positions [5]. Rotation may also occur due to foot and ankle positioning [7].

Many previous authors wondered if the rotation of the lower extremity affects alignment measurements on radiographs. Unfortunately, the simplest way to address this question—repeated radiographs of the same patient at different positions—is ethically problematic due to radiation exposure. Therefore, most previous studies targeted cadaveric legs or synthetic bones [6, 8–10, 12, 14, 15].

Some studies on the effect of rotation on radiographic measurements were performed on actual patients in an indirect manner using CT scans, but these studies have limitations in their clinical applicability. Kawakami et al. studied the outlines of 31 CT scans of medial osteoarthritis patients and calculated the maximum difference of the tibiofemoral angle (TFA) and hip-knee-ankle angle (HKAA) [5]. The study reported that the mean change in TFA and HKAA was 3.5° and 1.6° within the range of 8° of external rotation to 14° of internal rotation, respectively. However, this study targeted only TFA and HKAA and did not include other parameters. Jamali et al. analyzed CT scans for vascular work-up in normal populations using a virtual flat table in the computer environment and found that even a 3° rotational deviation can lead to a statistically significantly difference in the value of TFA and HKAA [4]. However, this study revealed only statistical significance without quantifying the difference, thus making it difficult to draw clinically applicable conclusions.

Furthermore, although there have been several studies on deformity models, no study has yet targeted real patients with deformities. Swanson et al. studied valgus and varus models using 3 saw bones with a plate and revealed that limbs with severe valgus or varus deformity were more sensitive to the effect of rotation [12]. Brouwer et al. demonstrated that rotation or flexion alone causes minimal changes in the projected angle, but when a varus knee flexes and rotates simultaneously, large changes occur in a flexion contracture model of a cadaveric leg [6].

To our knowledge, the effect of rotation in the measurement of radiologic alignment of the lower extremities has not been addressed in the knees of actual patients with deformities. Elucidating the rotational effect in patients with diverse types of deformity and quantifying the difference in angle are likely to improve patient classification and aid in choosing the most appropriate treatment option for each patient.

The objectives of this study were (1) to quantify the effect of lower extremity rotation on four common lower extremity alignment measurements, hip-knee-ankle angle (HKAA), lateral distal femoral angle (LDFA), medial proximal tibial angle (MPTA), and femur valgus angle (FVA); and (2) to compare these effects between groups of patients with varus, valgus, and flexion contracture deformity.

## 2. Materials and Methods

We retrospectively reviewed a total of 131 lower extremity 3D-computed tomography (3D-CT) scans of patients who visited our clinic with knee pain. The exclusion criteria included previous knee realignment surgery or hip arthroplasty. Of the 131 patients, 128 (56 males and 72 females) were included in this study. The average age of the patients was 56 years (range, 18–83).

To investigate the effect of flexion contracture and coronal alignment on angle measurement, the knees were categorized into the flexion contracture group (flexion contracture more than 10°), neutral group (HKAA between 175 and 185° and flexion contracture less than 10°), varus group (HKAA less than 175° and flexion contracture less than 10°), and valgus group (HKAA more than 185° and flexion contracture less than 10°). There were significant differences in sex and age and no differences in sides among the four groups (Table 1).

### 2.1. Digitally Reconstructed Radiographs

**Figure 1:**
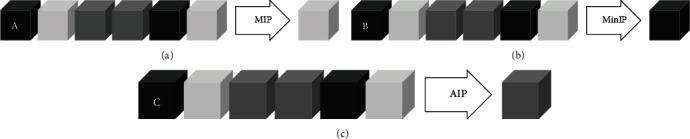
Models of three commonly used reconstruction algorithms. Each box indicates a voxel on the ray and a lighter box represents a higher Hounsfield value. Maximal intensity projection (MIP) represents the pixel with the highest Hounsfield number (a); the minimal intensity projection (MinIP) represents that with the lowest Hounsfield number (b); and the average intensity projection (AIP) represents the average (c).

To measure the mechanical axis of the lower extremities on radiographs, we reconstructed 2D virtual radiograms from 3D CT images. The simplest technique that is used to reconstruct 2D images from 3D images is to extract one single parameter of the volumetric data and produce two-dimensional (2D) reconstructions [16]. The most commonly used of these simple techniques are average intensity projection (AIP), maximal intensity projection (MIP), and minimal intensity projection (MinIP) (Figure 1). For each X-Y coordinate, MIP represents only the pixel with the highest Hounsfield number along the *z*-axis [16]. With this method, structures with lower attenuation are not visualized well. By contrast, MinIP cannot be used to visualize high-attenuation structures. Thus, we chose the AIP algorithm because we needed to see both high- and low-attenuation structures like the bone cortex and joint space to evaluate alignment.

**Figure 2:**
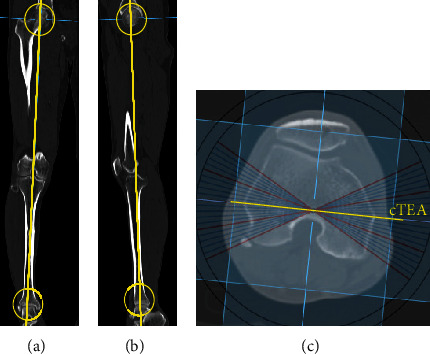
Creation of digitally reconstructed radiographs: reconstruction of 2D images from 3D CT images. The center of the femoral head to the center of the talus surface was set as the vertical axis in the coronal plane (a) and sagittal plane (b), and the clinical transepicondylar line was set as the horizontal axis (c).

We used the Xelis program (INFINITT Healthcare, Seoul, Republic of Korea) for 2D image reconstruction. With this program, we can freely set the axis and rotate the 3D image and convert the 3D image to 2D image. First, the weight-bearing line (a line drawn from the center of the femoral head to the center of the talus surface) was selected as the vertical axis and the clinical transepicondylar axis (cTEA) was chosen as the horizontal axis (Figure 2). Then, we set a plane formed by these two lines and the hypothetical rays were sent vertically to the plane. Averaging the voxels on the rays produced a digital X-ray reconstruction. We regarded this 2D image as the image of neutral rotation. And by rotating the hypothetical rays, we could obtain virtual 2D images at different rotational states with one 3D image at a fixed position.

**Figure 3:**
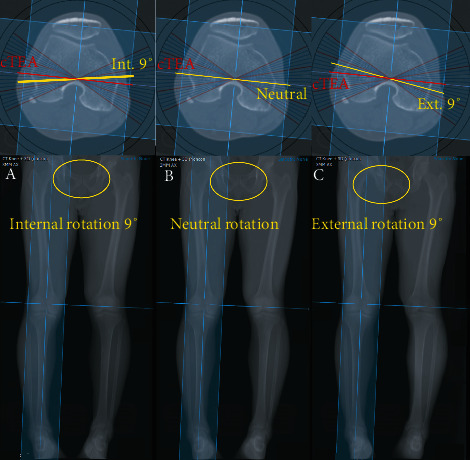
Examples of reconstructed images under different rotational states: 9° internal rotation (a); neutral position (b); and 9° external rotation (c).

To obtain a rotated image, a 3D-CT image was rotated on the vertical axis from internal 9° to external 9° in 3° increments and obtained images at various incidences of the hypothetical rays. In this way, seven 2D images of each virtual X-ray image (internal 9°, internal 6°, internal 3°, neutral, external 3°, external 6°, and external 9°) were obtained from each 3D-CT scan (Figure 3). Using these images, we measured the hip-knee-ankle angle (HKAA), lateral distal femoral angle (LDFA), medial proximal tibial angle (MPTA), and femoral valgus angle (FVA).

### 2.2. Statistical Analysis

Two orthopedic specialists measured the angles, and inter and intraobserver reliability analysis was performed using the intraclass correlation coefficient (ICC). The mean values of the angles were calculated for each parameter and analyzed within groups using a paired *t*-test. Evaluation of the differences between groups was done with one-way analysis of variance (ANOVA) with Tukey's method. Tukey's honestly significant difference (HSD) was used for post hoc analysis. All statistical analyses were performed using IBM SPSS Statistics 25 software (IBM Corp. Armonk, NY, USA) and Excel (Microsoft, Redmond, WA). Statistical significance was set at *P* < 0.05.

## 3. Results

**Figure 4:**
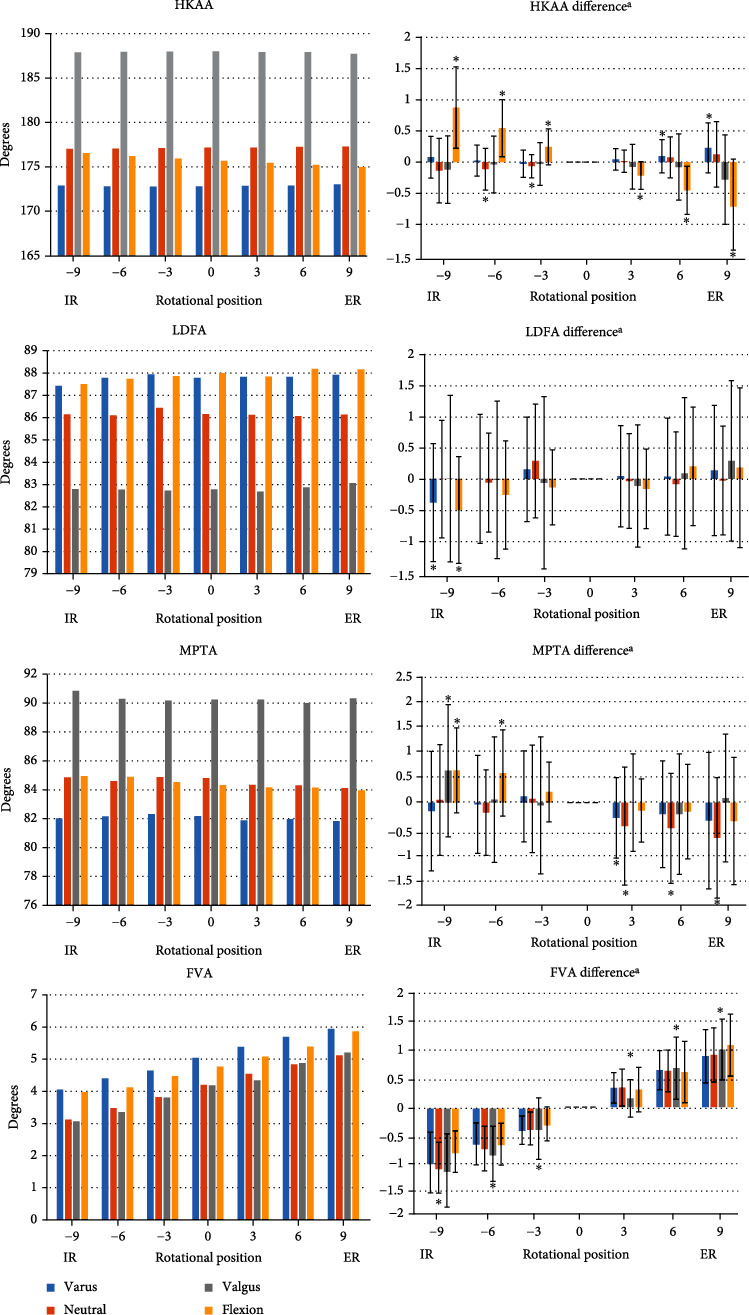
Radiographic angle measurements of the lower extremity under various degrees of rotation. ^a^Differences compared with neutral rotation. ^∗^Statistical significance of paired *t*-test compared to neutral rotation was set at *P* < 0.05.

FVA significantly differed according to the degree of rotation and showed a gradual, linear increasing pattern according to the degree of external rotation in all groups (*P* < 0.001). FVA increased by 0.90° under 9° external rotation and decreased by 0.98° under 9° internal rotation in the varus group; these numbers were 0.92°/-1.07° in the neutral group, 1.02°/-1.12° in the valgus group, and 1.10°/-0.79° in the flexion group. HKAA gradually decreased according to the degree of external rotation only in the flexion group (*P* < 0.001); it decreased by 0.71° under 9° external rotation and increased by 0.87° under 9° internal rotation. However, HKAA in the other groups, LDFA and MPTA was not significantly affected by rotation (Figure 4).

We next calculated the maximum difference in the measured angle within the 18° rotation range compared to the neutral rotation in each patient. The average of these maximal differences of FVA in all groups was 1.3° (±0.4°), and the average of the maximal differences of HKAA in the flexion group was 1.0° (±0.7°).

When comparing the differences between groups, only HKAA showed a significant difference in one-way ANOVA (*F* = 9.650, *P* < 0.001). The difference in HKAA in the flexion group was greater than that in the neutral, varus, and valgus groups. The other parameters showed no significant differences between groups (Table 2).

## 4. Discussion

The most important findings of this study are as follows: (1) rotation of the lower extremity affects radiographic angle measurements, especially FVA and HKKA, and (2) the effect of rotation on the measurement of HKKA was greater in the flexion group than in the other groups. These findings suggest that rotation of the lower extremity can lead to errors in angle measurement, especially the measurement of FVA and measurements taken in patients with flexion contracture.

Jamali et al., who analyzed 87 CT scans of normal patients taken for vascular work-up, found that, for TFA (tibiofemoral angle) and HKAA, even a 3° rotational deviation can lead to a significant difference in value [4]. Oswald et al. studied 38 cadaveric femurs and reported that external rotation will make the knees appear to have more varus angulation (0.2° per 5° of rotational deviation) [14]. Kawakami et al. found that the effect of rotation on limb alignment increased as the flexion angle increased in 31 CT scans of medial osteoarthritis patients [5]. Brouwer et al. studied 1 cadaveric leg at 3 positions (flexion 0°, 15°, and 30°) and reported that rotation or flexion alone causes minimal changes, but simultaneous flexion and rotation of the knee causes large changes [6]. Many studies have been done on saw bone models and cadaveric legs, which produced various results [8–10, 12, 15].

The common features of these previous studies were (1) rotation had a significant effect on FVA [12, 14] and (2) the effects were larger in the flexion group [5, 6]. The findings of the other parameters (HKAA, LDFA, and MPTA) were diverse. The present study revealed that the effect of rotation on FVA measurement was significant in all groups, and the average difference was 1.3° (±0.4°) within 18° of rotation. Additionally, in the flexion group, HKAA differed by 0.8° (±0.4°). These results are similar to those of previous studies, but our study had certain unique strengths: (1) we targeted real patients with diverse deformities, (2) we quantified the difference in angle measurements, and (3) we created conditions that were similar to those used for conventional X-ray-based angle measurement by reconstructing 2D X-ray images from 3D CT images.

Lee et al. reported that the femoral component varus malpositioning is the main origin of varus outliers and that the vulnerability of FVA measurement to rotation may lead to this result [17]. Thus, accurate angle measurements are essential.

### 4.1. Limitations

The main limitation of our study is that patients are placed in different positions for CT scans (supine) and conventional radiographs (standing). Since the change in angle is due to a change in joint space width, angles that do not cross the joint space such as FVA, MPTA, and LDFA are not affected by weight. [18] Brouwer et al. and Takehiko et al. reported an average of 2° varus deviation in the standing position [19, 20]. However, we targeted not the angle itself but the change in angle according to rotation. Furthermore, Jud et al. and Lazennec et al. analyzed the differences in HKA measurements between weight-bearing 2D images and non-weight-bearing 3D CT images and reported that the measurement of HKA in 2D images is more prone to measurement error [21, 22]. Therefore, positioning may be unimportant when interpreting the effect of rotation.

In addition, the reconstructed images used in this study are different from conventional plain X-ray images in that these virtual X-ray images do not demonstrate the parallax effect. On the other hand, our reconstructed 2D images may be more accurate due to the lack of the parallax effect. In addition, we rotated the images only in WBL, representing rotation of the legs, while rotation in multiple axes is possible in a clinical situation. Further studies of models with rotation in diverse axes may thus be useful.

## 5. Conclusion

Since rotation of the lower extremities can affect the alignment angle, it is necessary to check whether the patella is facing forward before diagnosing malalignment. As people with OA have various degrees of deformity, including flexion contracture, they are more vulnerable to rotation. The current study attempted to identify the effect of rotation on measurements of alignment in the lower extremities. The results suggest that knee surgeons should be careful and opt for more sensitive investigations when diagnosing and planning treatment options in certain groups of patients [23, 24].

## Figures and Tables

**Table 1:** Patient characteristics.

Variable	Varus (1) (*n* = 35)	Neutral (2) (*n* = 36)	Valgus (3) (*n* = 27)	Flexion (4) (*n* = 30)	*F*	*P*	Tukey HSD
	Mean ± SD or no						
Demographic							
Gender (male/female)	13/22	17/19	6/21	20/10		.006	
Rt/Lt	18/17	16/20	15/12	15/15		.848	
Age (years)	52.8 ± 12.2	54.5 ± 8.9	63.0 ± 16.2	55.4 ± 13.9	3.65	.015	1, 2 < 3
Radiographic angles(neutral rotation)							
HKAA (°)	172.79 ± 1.71	177.16 ± 2.04	188.00 ± 5.30	175.67 ± 4.61	99.66	<.001	1 < 2, 4 < 3
LDFA (°)	87.79 ± 2.16	86.15 ± 1.54	82.79 ± 3.62	87.99 ± 2.11	28.86	<.001	3 < 2 < 1, 4
MPTA (°)	82.18 ± 2.17	84.80 ± 2.05	90.23 ± 3.55	84.32 ± 3.12	46.41	<.001	1 < 2, 4 < 3
FVA (°)	5.04 ± 1.26	4.20 ± 1.30	4.19 ± 1.44	4.77 ± 1.66	3.00	.033	—

SD: standard deviation; HKAA: hip-knee-ankle angle; LDFA: lateral distal femoral angle; MPTA: medial proximal tibial angle; FVA: femoral valgus angle. The significance threshold for one-way analysis of variance (ANOVA) was set at *P* < 0.05. Tukey's honestly significant difference (HSD) was used for post hoc analysis. The significance threshold for Pearson's chi-square test was set at *P* < 0.05.

**Table 2:** Comparison of the effect of rotation on angle measurement between groups.

Classification		Difference of angle within 18° rotation			
		Mean ± SD	*F*	*P*	Tukey HSD
HKAA (°)	Neutral (1)	0.52 ± 0.33	9.650	<0.001	4 > 1, 4 > 2
	Varus (2)	0.48 ± 0.24			4 > 3, 3 > 2
	Valgus (3)	0.80 ± 0.40			
	Flexion (4)	0.99 ± 0.69			
LDFA (°)	Neutral	1.35 ± 0.47	0.890	0.448	
	Varus	1.36 ± 0.52			
	Valgus	1.56 ± 0.74			
	Flexion	1.31 ± 0.76			
MPTA (°)	Neutral	1.72 ± 0.66	1.723	0.166	
	Varus	1.69 ± 0.69			
	Valgus	1.84 ± 0.79			
	Flexion	1.43 ± 0.69			
FVA (°)	Neutral	1.30 ± 0.39	1.238	0.299	
	Varus	1.28 ± 0.41			
	Valgus	1.45 ± 0.49			
	Flexion	1.24 ± 0.40			

SD: standard deviation; HKAA: hip-knee-ankle angle; LDFA: lateral distal femoral angle; MPTA: medial proximal tibial angle; FVA: femoral valgus angle. The significance threshold for one-way analysis of variance (ANOVA) was set at *P* < 0.05. Tukey's honestly significant difference (HSD) was used for post hoc analysis.

## Data Availability

The data generated during the current study are available from the corresponding author upon reasonable request.
